# Two dimensional analysis of low pressure flows in the annulus region between two concentric cylinders

**DOI:** 10.1186/s40064-016-2140-6

**Published:** 2016-04-26

**Authors:** Wael Al-Kouz, Aiman Alshare, Ammar Alkhalidi, Suhil Kiwan

**Affiliations:** Mechatronics Engineering Department, German Jordanian University, Amman, Jordan; Mechanical and Maintenance Engineering Department, German Jordanian University, Amman, Jordan; Energy Engineering Department, German Jordanian University, Amman, Jordan; Mechanical Engineering Department, Jordan University of Science and Technology, Irbid, Jordan

**Keywords:** Natural convection, Heat transfer, Low pressure, Concentric cylinders, Solar collectors, Parabolic trough

## Abstract

A numerical simulation of the steady two-dimensional laminar natural convection heat transfer for the gaseous low-pressure flows in the annulus region between two concentric horizontal cylinders is carried out. This type of flow occurs in “evacuated” solar collectors and in the receivers of the solar parabolic trough collectors. A finite volume code is used to solve the coupled set of governing equations. Boussinesq approximation is utilized to model the buoyancy effect. A correlation for the thermal conductivity ratio (*k*_r_ = *k*_eff_/*k*) in terms of Knudsen number and the modified Rayleigh number is proposed for Prandtl number (*Pr* = 0.701). It is found that as Knudsen number increases then the thermal conductivity ratio decreases for a given Rayleigh number. Also, it is shown that the thermal conductivity ratio *k*_r_ increases as Rayleigh number increases. It appears that there is no consistent trend for varying the dimensionless gap spacing between the inner and the outer cylinder ($\overline{{L_{g} }}$) on the thermal conductivity ratio (*k*_r_) for the considered spacing range.

## Background

Gaseous rarefied flows in microscale geometries have been studied extensively in the past 20 years due to their relevance to micromachined and MEMS devices or sensors. Additional research activities in this field was driven by the wide application of such devices and sensors in the aerospace industry, biomedical engineering, plasma applications in material processing (Karniadakis et al. 2005; Bird 1994), latent energy storage systems and transmission cable cooling systems. One important application for rarefied gaseous flows is found in the parabolic trough collectors (PTCs) used in solar power plants. Heat transfer analysis for PTCs plays an important role in determining heat losses and efficiencies of the power plant (Patnode 2006). The problem rises in such application when complete evacuation in solar receivers is assumed. In fact, complete evacuation is either difficult or impractical to achieve. In many cases, complete evacuation adds limitations on material used to fabricate the receiver (glass in solar energy applications). When complete evacuation is assumed, the convection heat transfer is zero, while, if complete evacuation is not achieved, the value of the convection heat transfer is not zero. This value depends on the pressure inside the space.

Another issue related to receivers in PTC is to determine the range of “low pressure” inside the receiver that minimizes the heat transfer losses. This pressure is a related to the operating temperature and the mean free path (Karniadakis et al. 2005; Bird 1994):

1 $$\lambda = \frac{{k_{B} T}}{{\sqrt 2 \pi d^{2} P}}$$

where $k_{B}$ is the Boltzmann constant, *T* the temperature, *P* the pressure, *d* is molecular diameter of the gas under investigation and $\lambda$ is the mean free path.

The main aim of this study is to establish and present the correlation between the heat transfer and the pressure in the space is a simple form. Such correlation will facilitate conducting the heat transfer calculations for design engineers.

Rarefied, micro and nano scale flows are characterized using the dimensionless Knudsen number ($Kn$). This number is the ratio of the mean free path ($\lambda$) to the characteristic length ($L$) of the geometry of interest. It measures the degree of rarefaction of the flow. Based on the Knudsen number, the respective flow regimes are classified into four types according to Schaaf and Chambre (1961) and Cercignani and Lampis (1974). For $Kn < 0.01$ the flow is in the continuum regime in which the Navier–Stokes equations are used to describe the flow. If $0.01 < Kn < 0.1$, the flow is in the slip regime in which the Navier–Stokes equations are used with velocity slip and temperature jump boundary conditions to describe the flow. Similarity method can be used to solve for the convection heat transfer in the slip flow regime; one example is the convection heat transfer over linearly stretched isothermal microsurface that was investigated by Kiwan and Al-Nimr (2009). They presented correlations for skin friction coefficient and Nusselt number in terms of velocity slip and temperature jump parameters. Kiwan and Al-Nimr (2010) also showed that complete similarity solution is possible for boundary layer flows only for a stagnation flow over isothermal microsurfaces. They found that skin friction coefficient is inversely proportional to both the slip velocity parameter and local Reynolds number. In the range of $0.1 < Kn < 10$, the flow is in the transitional regime and for $10 < Kn$, the collision between particles is very rare and the flow is in the free molecular regime. Flow characteristics for transitional and free molecular regimes are solved primarily utilizing particle simulation methods such as the direct simulation Monte Carlo (DSMC) method. For instance, the supersonic gaseous flows into nanochannels using the unstructured 3-D direct simulation Monte Carlo method is investigated by Gatsonis et al. (2010). In their study, slip, transitional and free molecular regimes are been investigated. They found that the flow and heat transfer characteristics are affected by inlet Mach number (*Ma*), inlet pressure and the aspect ratio of the channel.

The flow and heat transfer for similar shapes (Annulus region between two concentric cylinders) in no-slip flows have been extensively studied and documented. A comprehensive literature review for the numerical and experimental investigations for the flow in the region of the annuli between two concentric cylinders was given by (Kuehn and Goldstein 1974; Kuehn and Goldstein 1976).

Free convection heat transfer in the annular space between long horizontal concentric cylinders are considered by Raithby and Hollands (1975). In their study, they presented a correlation for the conductivity ratio that is valid for $0.7 \le \Pr \le 6000$ and $Ra_{c} \le 10^{7}$. Their correlation is given as follows:

2 $$k_{r} = \frac{{k_{eff} }}{k} = 0.386\left( {\frac{\Pr }{0.861 + \Pr }} \right)^{1/4} Ra_{c}^{1/4}$$

where *k*_*eff*_ is a fictitious thermal conductivity for a stationary fluid that will transfer the same amount of heat as the actual moving fluid, and *k* is the thermal conductivity of the fluid at atmospheric pressure. Whereas, the length scale $L_{c}$ is given as follows:

3 $$L_{c} = \frac{{2\left[ {\ln \left( {r_{ \circ } /r_{i} } \right)} \right]^{4/3} }}{{\left( {r_{i}^{ - 3/5} + r_{ \circ }^{ - 3/5} } \right)^{5/3} }}$$

The free convection in the annulus region between two concentric cylinders is investigated by Mack and Bishop (1968). They used a truncated power series in terms of Rayleigh number to represent the stream function and the temperature variables. A summary for the experimental work and a correlation (using the conduction boundary layer model) to the flow in the annulus between two concentric horizontal cylinders are presented by (Kuehn and Goldstein 1974; Kuehn and Goldstein 1976). El-Sherbiny (2004) studied numerically the effect of Rayleigh number (10^2^–10^6^) and the radii ratio (1.25 and 10) on the characteristics of the flow in the annuli of two infinite concentric cylinders.

The effect of *Ra*, *Pr*, the inclination angle as well as the thermal conductivity ratio on the velocity and temperature fields of a laminar natural convection in an inclined cylindrical enclosure having finite thickness walls was investigated by Sheremet (2012). The study showed that it is possible to indicate two intervals with a maximum magnitude of the generalized heat transfer coefficient at various values of the inclination angle of the tube. Furthermore, it is found that when Rayleigh number is less than 1 × 10^5^, the thermal component of the natural convection is dominant, while, when Rayleigh number is greater than 1 × 10^5^, then the hydrodynamic component of natural convection is dominant.

In the work done by Fattahi et al. (2010), mixed convection heat transfer in eccentric annulus was simulated numerically by lattice Boltzmann model (LBM). The effect of eccentricity on heat transfer at various locations was examined at *Ra* = 10^4^ and annulus gap width ratio of 2. Velocity and temperature distributions as well as Nusselt number are obtained. It was shown that heat transfer improves when the inner cylinder moves downward regardless of the radial position.

The effect of fin conductivity ratio, Darcy number and Rayleigh number on the heat transfer characteristics for porous fins attached to the inner cylinder of the annulus between two concentric cylinders is investigated by Kiwan and Zeitoun (2008). They found reported enhancement in the heat transfer by using porous fins. They also reported that with porous fin, unlike the solid fins, the heat transfer decreases by increasing the inclination angle of the fin inside the annulus. Ghernoug et al. (2013) numerically studied the effect of Grashof number on the natural convection characteristics in the annular space between two eccentric horizontal cylinders. They found that conduction heat transfer is dominant for the case when Grashof number is less than 5 × 10^4^. Whereas, for larger values Grashof number, the convection heat transfer is dominant.

In the work carried out by Bouras et al. (2013), velocity stream function formulation with Boussinesq approximation are used to investigate the effect of Prandtl number and Rayleigh number on the natural convection in the annulus space between two elliptical confocal cylinders. They concluded that for low Rayleigh numbers, there is no effect for Prandtl number on the heat transfer. While increasing Prandtl number increases the heat transfer at higher Rayleigh number flows.

Bouras et al. (2014) numerically investigated the double diffusive natural heat transfer convection in the annular space between confocal elliptic shape enclosures. It was found that both heat and mass transfer increase with increasing Rayleigh number. At large Rayleigh numbers, the iso-concentrations exhibit a plume similar to isotherms. However, it was found that this plume diffuses throughout the annular space when Lewis number is greater than one.

Cianfrini et al. (2011) investigated the natural convection heat transfer of nanofluids in annular spaces between horizontal concentric cylinders, two empirical equations based on a wide variety of experimental data are used for evaluation of the nanofluid effective thermal conductivity and dynamic viscosity, while the other effective properties are calculated based on the mixing theory. The heat transfer enhancement due to the nanoparticles dispersion in the liquid is calculated for different conditions, such as the diameter of the particles. It is concluded that there exist an optimum particle loading corresponding to the maximum heat transfer. In the numerical study conducted by Chmaissem et al. (2002) for the natural convection heat transfer in annular space, it is reported that the enclosure impedes movement of the fluid and there is a possibility to develop a multi-cellular regions even if Rayleigh number is small.

Padilla et al. (2011) analyzed the heat transfer of the parabolic trough solar receiver and presented correlations for the heat transfer coefficients. They used a receiver of inner diameter of 70 mm and outer diameter is 115 mm. Price et al. (2002) and Thomas (1979) investigated the flow in the parabolic trough solar collector receiver. They evacuated the annulus to pressure less than one Torr. This operating pressure range is within the so called free molecular regime in which collisions between particles are very rare. They found out that the resulting pressure for the free molecular regime in which Knudsen number is greater than 10 is approximately 0.013 Pa. The heat transfer coefficient of the flow in the annulus between two horizontal cylinders for the pressures that is less than 1 Torr or Free molecular regime, is derived and given by Dushman (1962). While for the case where the pressure is greater than 1 Torr, the conduction layer model has shown to be able to predict the heat transfer Rohsenow et al. (2006).

In this work, a finite volume numerical technique utilizing Boussinesq approximation is used to obtain the solution for the natural convection heat transfer characteristics between two concentric horizontal cylinders. The software package (FLUENT 16) is used to conduct the simulations. The inner cylinder is subjected to a higher temperature than the outer cylinder. Prandtl number (*Pr*) is taken to be constant and is equal to 0.701. Effects of Knudsen number (*Kn*), modified Rayleigh number (*Ra*_m_) and the annulus gap spacing on the flow and heat transfer characteristics is investigated and documented.

## Methods

The problem under consideration is treated as steady-state, two dimensional, and laminar flow. All fluid properties are considered constant except the density where Boussinesq approximation is applied to account for the buoyancy force. Flow Slip and temperature jump boundary conditions are imposed at the fluid-solid interface.

**Fig. 1:**
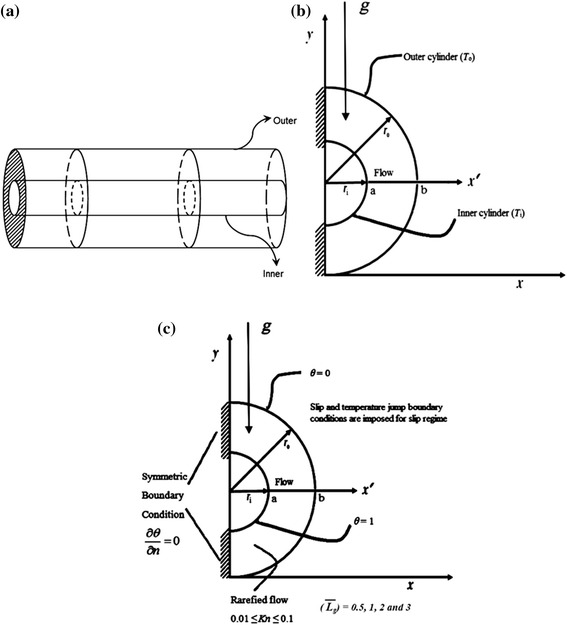
**a** Parabolic trough receiver. **b** The geometry used for the computational domain, the annular regime between two concentric cylinders. **c** Geometry and boundary conditions integrated to the computational domain, the annular regime between two concentric cylinders

Figure 1a shows the receiver used in the parabolic trough collectors, which is one of the most important industrial applications, that relates to our study. Figure 1b illustrates the computational domain in the annulus region between the two concentric cylinders. Slip flow regime in which Knudsen number is greater than 0.01 and less than 0.1 is investigated.

The governing equations that describe the problem are summarized below:

Continuity:

4 $$\frac{\partial u}{\partial x} + \frac{\partial v}{\partial y} = 0$$

*x*-momentum:

5 $$\rho u\frac{\partial u}{\partial x} + \rho v\frac{\partial u}{\partial y} = - \frac{\partial P}{\partial x} + \mu \left[ {\frac{{\partial^{2} u}}{{\partial x^{2} }} + \frac{{\partial^{2} u}}{{\partial y^{2} }}} \right]$$

Note that the *x* component of the gravity is equal to zero.

*y*-momentum:

6 $$\rho u\frac{\partial v}{\partial x} + \rho v\frac{\partial v}{\partial y} = - \frac{\partial P}{\partial y} - \rho g_{y} + \mu \left[ {\frac{{\partial^{2} v}}{{\partial x^{2} }} + \frac{{\partial^{2} v}}{{\partial y^{2} }}} \right]$$

Energy:

7 $$\rho C_{p} u\frac{\partial T}{\partial x} + \rho C_{p} v\frac{\partial T}{\partial y} = k\left[ {\frac{{\partial^{2} T}}{{\partial x^{2} }} + \frac{{\partial^{2} T}}{{\partial y^{2} }}} \right]$$

To estimate the fluid density, the ideal gas state equation is provided as an input,

8 $$\rho = \frac{P}{RT}$$

The boundary conditions associated with the governing equations for the problem are: Velocity slip and temperature jump at the cavity walls; reported by Karniadakis et al. (2005), Lockerby et al. (2004) and Colin (2006) as follows

9a $$u_{w} - u_{g} = \left( {\frac{{2 - \sigma_{v} }}{{\sigma_{v} }}} \right)\lambda \frac{\partial u}{\partial n} \approx \left( {\frac{{2 - \sigma_{v} }}{{\sigma_{v} }}} \right)K_{n} \left( {u_{g} - u_{c} } \right)$$

where $u_{c}$ is the tangential velocity of the first cell from the wall in the computational domain.

9b $$v_{g} = 0$$

9c $$T_{w} - T_{g} = \left( {\frac{{2 - \sigma_{T} }}{{\sigma_{T} }}} \right)\frac{2\gamma }{\gamma + 1}\frac{k}{{\mu c_{v} }}\lambda \frac{\partial T}{\partial n} \approx \left( {\frac{{2 - \sigma_{T} }}{{\sigma_{T} }}} \right)\frac{2\gamma }{\gamma + 1}\frac{k}{{\mu c_{v} }}K_{n} \left( {T_{g} - T_{c} } \right)$$

where *T*_*c*_ is temperature of the first cell from the wall in the computational domain.

In Eqs. (9a) and (9c), $\sigma_{v}$ and $\sigma_{T}$ represent the momentum and thermal accommodation coefficients and used as an inputs in the simulations, where:

10 $$\sigma_{v} = \frac{{\tau_{i} - \tau_{r} }}{{\tau_{\text{i}} - \tau_{\text{w}} }}$$

where $\tau_{i}$ represents the tangential momentum of incoming particles to a certain surface (wall) and $\tau_{r}$ represents the tangential momentum of the reflected particles from that surface. While, $\tau_{\text{w}}$ is the tangential momentum of reemitted molecules from the surface with a temperature equal to the surface (wall) temperature (Karniadakis et al. 2005).

11 $$\sigma_{T} = \frac{{dE_{i} - dE_{r}^{{}} }}{{dE_{i} - dE_{w} }}$$

where *dE*_*i*_ is the energy flux of the incoming particles on a surface per unit time, *dE*_*r*_ represents the energy flux of the reflected particles per unit time, and *dE*_*w*_ denotes the energy flux of all the incoming particles that had been reemitted with the energy flux corresponding to the surface temperature *T*_*w*_.

The corresponding Knudsen number (*Kn*) is defined as follows:

12 $$Kn = \frac{\lambda }{{L_{g} }}$$

where *L*_g_ is the gap spacing between the inner and outer cylinders

Let’s define the dimensionless temperature *θ*, where

13 $$\theta = \frac{{T - T_{ \circ } }}{{T_{i} - T_{ \circ } }}$$

The radial boundary conditions are imposed as follows:

14 $${\text{At}}\quad r = r_{i} ,\;T = T_{i} ,\quad \theta = 1$$

15 $${\text{At}}\quad r = r_{o} ,\;T = T_{o} ,\quad \theta = 0$$

where *T*_i_ is higher than *T*_o_. Figure 1c shows the geometry under investigation integrated with the boundary conditions.

The modified Rayleigh number was introduced by Alshahrani and Zeitoun (2005a, b) as:

16 $$Ra_{m} = Ra_{i}^{1/4} \left( {0.1389\left( {1 - \frac{{D_{i} }}{{D_{ \circ } }}} \right) + 0.0927} \right)\ln \left( {\frac{{D_{ \circ } }}{{D_{i} }}} \right)$$

The modified Rayleigh number absorbs the geometrical effects that are related to the flow in the annulus region of two concentric horizontal cylinders.

The values of $k_{r}$ are calculated as follows:

The local heat flux at the inner cylinder wall is calculated using Fourier’s law of conduction,

17 $$q_{i} = - k\frac{\partial T}{\partial n}$$

Since the problem is steady, the heat transfer from the inner to the outer wall is calculated by integrating the local heat flux along the wall of the inner cylinder,

18 $$Q = \int\limits_{{A_{i} }} {q_{i} dA}$$

Then, the average heat transfer coefficient along the wall of the inner cylinder is calculated from:

19 $$\bar{h}_{i} = \frac{Q}{{(T_{i} - T_{ \circ } )A_{i} }}$$

The average Nusselt number is calculated from

20 $$\overline{{Nu_{i} }} = \frac{{\bar{h}_{i} D_{i} }}{k}$$

The conduction heat transfer in cylindrical annuli for a stagnant fluid is obtained as follows:

21 $$Q_{cond} = \frac{{2\pi kL(T_{i} - T_{ \circ } )}}{{\ln (D_{ \circ } /D_{i} )}}$$

The combined effect of both conduction and convection in the annulus region is calculated as follows:

22 $$Q = \frac{{2\pi k_{eff} L(T_{i} - T_{ \circ } )}}{{\ln (D_{ \circ } /D_{i} )}}$$

Combining the above equations to obtain the expression:

23 $$k_{r} = \frac{{k_{eff} }}{k} = \overline{{Nu_{i} }} \frac{{\ln \left( {{{D_{o} } \mathord{\left/ {\vphantom {{D_{o} } {D_{i} }}} \right. \kern-0pt} {D_{i} }}} \right)}}{2}$$

The method of solution used in this study is based on a finite volume analysis. The SIMPLE algorithm adopted from Versteeg and Malalasekera (1995) and Patankar and Spalding (1972) is used to calculate the pressure field. To differentiate the convective terms, a hybrid scheme, which is a combination of the central difference, and upwind difference schemes is being used; the scheme is second order accurate. The discretized equations are solved and the iterative solution is considered to be converged when the maximum of the normalized absolute residual across all nodes is less than 10^−6^.

**Fig. 2:**
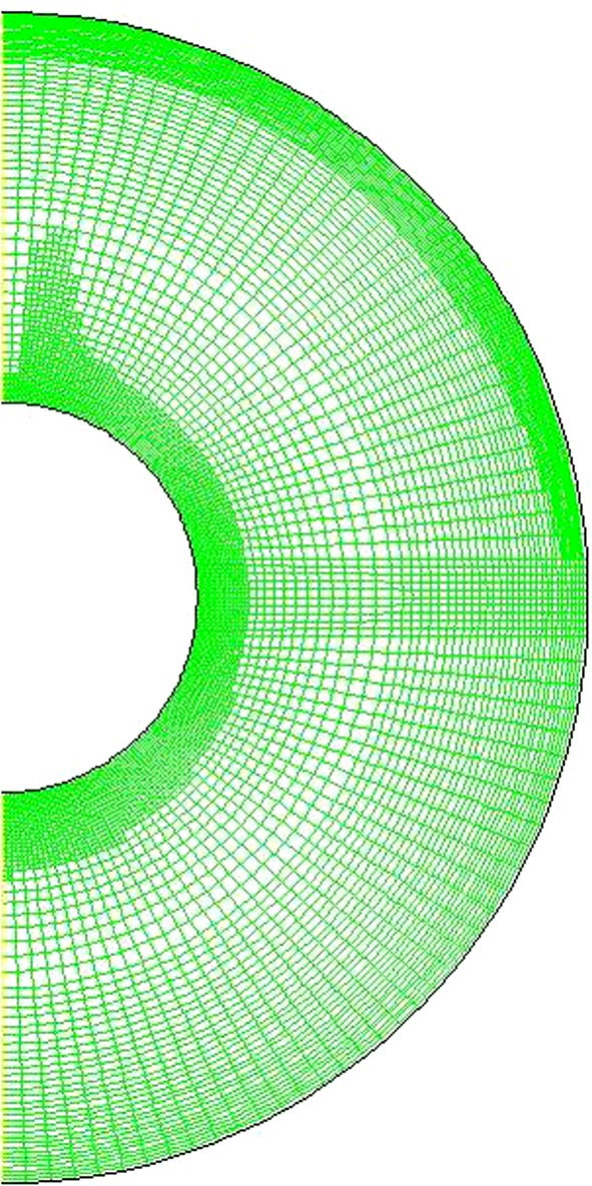
Adaptive grid system technique used in the simulations

 Figure 2 shows a sample of computational mesh for the domain under consideration. For the starting mesh, the grid step sizes are increasing in the radial and azimuthal directions with expansion factors of 1.06 and 1.15 respectively. A grid independency test is carried out by monitoring the heat transfer per unit length. The number of grid nodes are increased until a point is reached where the solution does not change with further mesh refinement. The results are summarized in Table 1 when $Ra = 5.3 \times 10^{4}$. It is clear from the table that the solution is mesh-independent for a grid of 80 × 480 in the radial and azimuthal directions, respectively. This grid size is used for all cases of *Ra*.

**Table 1:** Grid independent study

Mesh	$Q^{\prime}$ (W/m)	Percentage change compared to 80 × 480 mesh
40 × 360	44.924	2.02
60 × 360	44.239	0.46
80 × 360	43.989	−0.10
80 × 480	44.034	0

**Fig. 3:**
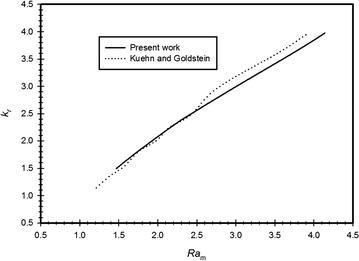
Comparison between current data and (Kuehn and Goldstein 1974)

To verify the numerical code, the results of the present code are tested and compared with the results obtained by (Kuehn and Goldstein 1974; Kuehn and Goldstein 1976). The natural convection heat transfer of the steady state, laminar flow in a horizontal cylindrical annulus is solved numerically. Figure 3 illustrates a comparison between $k_{r} = \frac{{k_{eff} }}{k}$ obtained by the present code and that obtained by (Kuehn and Goldstein 1974; Kuehn and Goldstein 1976); it should be noted that the value of $k_{eff}$ is based on the integrated heat transfer from the whole tube, the verification utilizes $Ra = 5.3 \times 10^{4}$ and an enclosure aspect ratio of 5. The comparison is excellent with maximum error less than 1.3 %. It should be noted that (Kiwan and Khodier 2008) presented other validation studies involving porous medium using the same code where they simulated the steady-state, laminar, two-dimensional, natural convection heat transfer in an open-ended channel partially filled with an isotropic porous medium.

## Results and discussion

The effects of varying modified Rayleigh number (*Ra*_m_), Knudsen number (*Kn*) and the spacing between the outer and inner cylinders (*L*_g_) on the thermal conductivity ratio are investigated. The results of these investigations will be presented and discussed next.

**Fig. 4:**
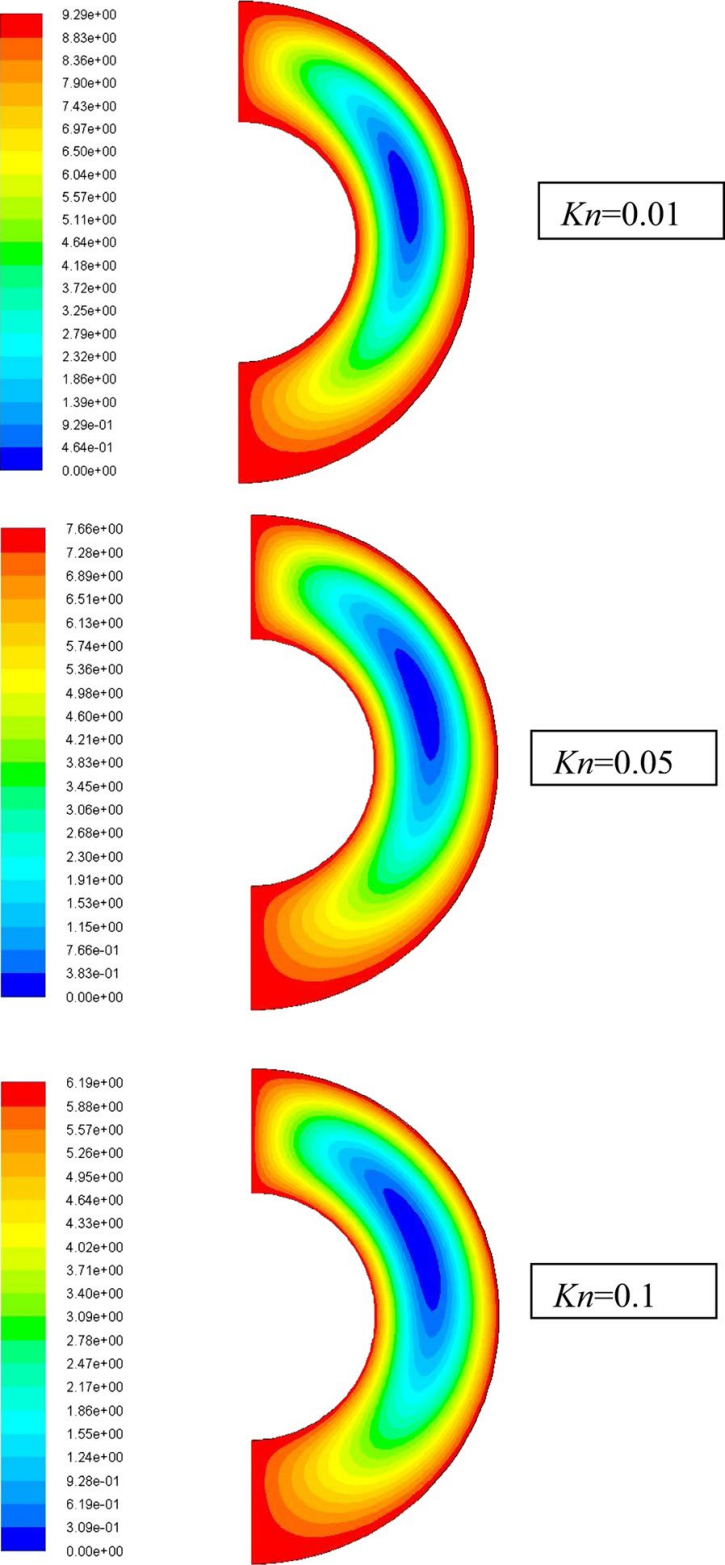
Streamlines (dimensionless gap spacing $\overline{{L_{g} }}$ = 2, *Ra*_m_ = 3.86)

The velocity stream function contours for $\overline{{L_{g} }}$ = 2, *Ra*_m_ = 3.86 and different values of *Kn* are shown in Fig. 4. It is clear that for all cases a rotational cell is formed in the annulus spacing. The strength and location of this cell depend on the value of *Kn*. It is clear from the graph that as Knudsen number increases, the strength of the rotational cell decreases and its center shifts downward. This can be attributed to the rarefaction effect; the increase in rarefaction will increase the slip velocity at the walls. This reduces the interaction between molecules and thus reduces their velocities.

**Fig. 5:**
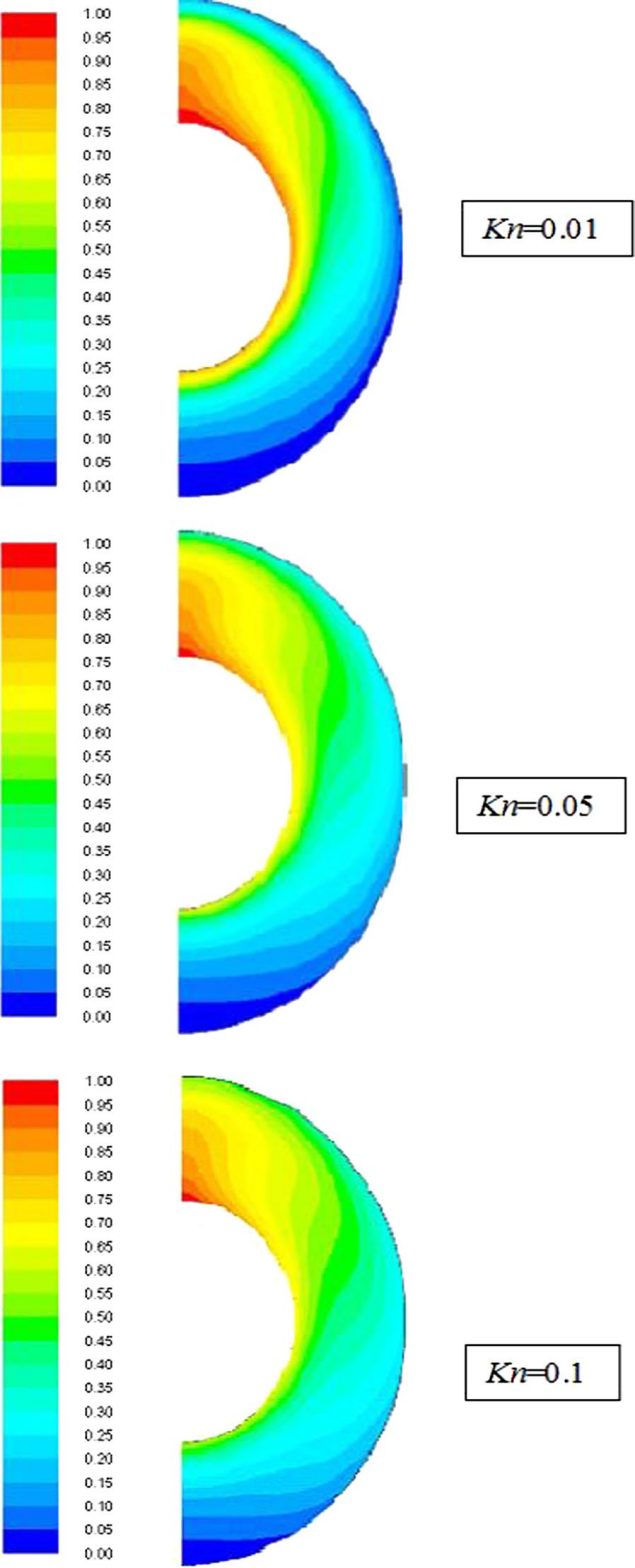
Isotherms (dimensionless gap spacing $\overline{{L_{g} }}$ = 2, *Ra*_m_ = 3.86)

The isotherms of the dimensionless temperature for $\overline{{L_{g} }}$ = 2, *Ra*_m_ = 3.86 and different values of *Kn* are drawn in Fig. 5. The contours show that for all cases, the lower part of the annuli represents a case of a dominant conduction mode of heat transfer, while, in the upper part of the annulus (70–90 degree CW from the centerline) the convection mode is dominant. This can be attributed to the recirculating fluid driven by the buoyancy effect. In the region of 0 and 30 degrees counter clockwise from the centerline, the effect of convection heat transfer is minor. It is also clear from the graph that as Knudsen number increases then the distortion and mixing of the flow decreases and consequently the heat transfer decreases.

**Fig. 6:**
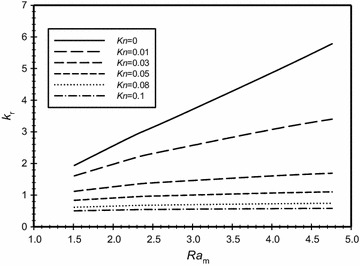
Variation of the effective conductivity ratio for different Knudsen numbers for the case of the dimensionless gap spacing $\overline{{L_{g} }}$ = 3 for different modified Rayleigh numbers

The effect of modified Rayleigh number on the conductivity ratio (*k*_r_) for a range of Knudsen number that covers both slip and no-slip cases for the case of $\overline{{L_{g} }}$ = 3 is illustrated in Fig. 6. It is seen in the graph that at fixed values of Ra_m_, as *Kn* increases then the value of the conductivity ratio decreases. This can be explained as follows: when Knudsen number increases then the temperature jump at the wall increases (see Eq. 9c). This will reduce the temperature of the gas adjacent to the wall and, therefore, will reduce the temperature difference across the gap resulting in reducing the heat transfer from the inner to outer wall. The figure also shows that as the modified Rayleigh number increases for the same Knudsen number then the value of the conductivity ratio increases. This indicates that the convection mode of heat transfer becomes more effective, however, the effect of varying the modified Rayleigh number on *k*_*r*_ diminishes and becomes of a small value as Knudsen number increases beyond *Kn* = 0.05. It should be noted that the value of the conductivity ratio is less than one for Knudsen number values greater than 0.05. This does not mean that the heat transfer value is less than the value of heat transfer by conduction. This happens because the value of *k* used in defining *k*_*r*_ is taken at atmospheric pressure as a reference value. Thus, when *k*_*r*_ is less than one, it means that the heat transfer by convection is less than the heat transfer by conduction in a fluid having the reference value of *k*.

Other cases where the values of $\overline{{L_{g} }}$ = 1 and 2 were tested and the results were very similar to those of the case of $\overline{{L_{g} }}$ = 3.

**Fig. 7:**
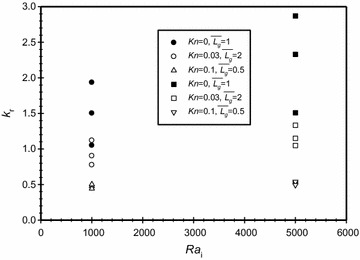
Variation of the effective conductivity ratio for different Knudsen numbers for *Ra*_i_ = 5×10^3^, *Ra*_i_ = 1×10^3^ and $\overline{{L_{g} }}$ = 1, 2 and 0.5

To isolate the geometrical effects ($\overline{{L_{g} }}$) included in the definition of *Ra*_m_, Fig. 7 is drawn. It shows the effect of varying *Kn* on *k*_*r*_ for two values Rayleigh number based on the inner diameter, *Ra*_i_ = 1 × 10^3^ and 5 × 10^3^ and different gap spacing ($\overline{{L_{g} }}$). It is clear from the graph that there is no consistent trend of varying the gap spacing on the conductivity ratio for the investigated range of gaps; this can be explained as one can get the same spacing by changing the inner or the outer diameters of the annuli. The graph also shows that as Knudsen number increases then the conductivity ratio decreases. However, the effect of varying Knudsen number on the conductivity ratio diminishes as Knudsen number increases. This graph also shows that for higher Knudsen numbers, the basic mode of heat transfer is conduction heat transfer. The graph also shows that the conductivity ratio is below one for certain ranges of Knudsen numbers as explained earlier.

A correlation for the conductivity ratio (*k*_r_) as a function of Knudsen number (*Kn*) and the modified Rayleigh number (*Ra*_m_) is proposed. The correlation is obtained using the least square regression using the simulation results. This correlation takes the following form:

24 $$k_{\text{r}} = \frac{{k_{eff} }}{k} = 0.0987\left( {Kn} \right)^{ - 0.619} Ra_{m}^{1/4}$$

It is obvious from Eq. (24) that the conductivity ratio is directly proportional to $Ra_{m}^{1/4}$ and inversely proportional to $\left( {Kn} \right)^{0.619}$. It should be noted that Eq. (24) is only applicable for the slip regime in which *Kn* is greater than 0.01 and less than 0.1 and for a fixed *Pr* of 0.701.

**Fig. 8:**
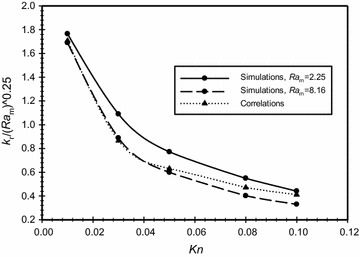
Comparison between the correlations and the simulation results for the effective conductivity ratio as a function of Knudsen number for $\overline{{L_{g} }}$ = 3

Figure 8 shows a comparison between the simulation and the correlation results of the values of $\frac{{k_{r} }}{{Ra_{m}^{1/4} }}$ as a function of Knudsen number. Modified Rayleigh number was varied to take the values of 2.25 and 8.16 while the dimensionless gap spacing was fixed ($\overline{{L_{g} }}$ = 3). It is obvious from the graph that the curves are almost identical for Knudsen number values less than 0.05 and with an acceptable error of about 13 % for higher Knudsen number values.

**Fig. 9:**
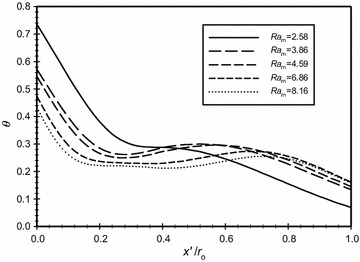
Variation of the dimensionless temperature for different dimensionless axial distance for $\overline{{L_{g} }}$ = 3, *Kn* = 0.05

The dimensionless temperature is plotted in Fig. 9 versus the dimensionless axial distance ($\overline{X}$) for various modified Rayleigh numbers (*Ra*_m_). It is shown in the figure that as *Ra*_m_ increases then the dimensionless temperature at the inner boundaries decreases. This results in decreasing the amount of heat transfers to the annular space. It is also seen in the graph and for the same modified Rayleigh number, the variation of the dimensionless temperature increases in wall boundary regions and nearly constant in the middle region of the annulus.

**Fig. 10:**
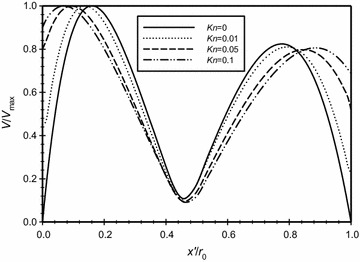
Variation of the dimensionless velocity magnitude for different dimensionless axial distance for $\overline{{L_{g} }}$ = 2, *Ra*_m_ = 2.25

**Fig. 11:**
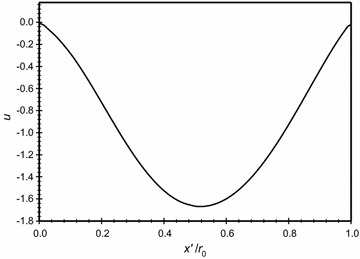
Variation of the *x* velocity for different dimensionless axial distance for $\overline{{L_{g} }} = 2$, *Ra*_m_ = 2.25

**Fig. 12:**
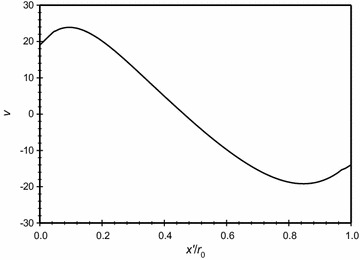
Variation of the *y* velocity for different dimensionless axial distance for $\overline{{L_{g} }} = 2$, *Ra*_m_ = 2.25

The distribution of the dimensionless velocity magnitude along a horizontal centerline in the annulus space is plotted in Fig. 10 for different *Kn* that is covering the no-slip and slip boundary conditions, *Ra*_m_ = 2.25, and $\overline{{L_{g} }}$ = 2. It is obvious from the figure and as expected, increasing Knudsen number will increase the velocity slip at the boundaries. In addition to that, in the middle region away from the boundaries the velocity drops and, consequently, this makes the conduction mode to be dominant. The *x* velocity distribution along the centerline of the annuli for the same case is plotted in Fig. 11. While the *y* velocity component is plotted in Fig. 12. The graphs demonstrates the presence of a convection cell in the annulus: the flow is upward close to the inner cylinder (hotter cylinder) and it is downward near the outer cylinder.

**Fig. 13:**
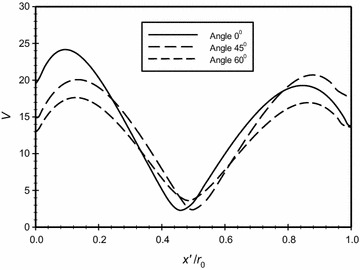
Variation of the velocity magnitude for different dimensionless axial distance at different angles from the center point of the annuli for $\overline{{L_{g} }} = 2$, *Ra*_m_ = 2.25

**Fig. 14:**
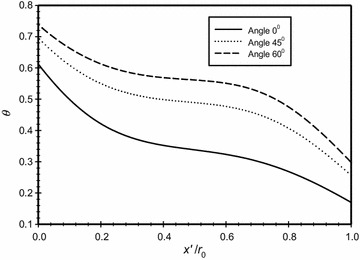
Variation of the dimensionless temperature for different dimensionless axial distance at different angles from the center point of the annuli for $\overline{{L_{g} }} = 2$, *Ra*_m_ = 2.25

Figures 13 and 14 show respectively the velocity magnitude and the dimensionless temperature distributions along three different lines that correspond to three different angles from the center of the annuli; namely (0°, 45° and 60°). The graphs clearly show the slip and the temperature jump at the boundaries for the three different cases.

**Fig. 15:**
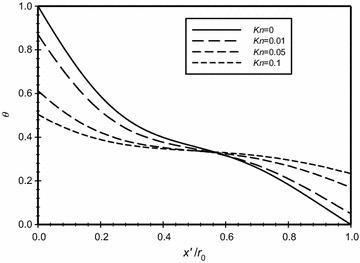
Variation of the dimensionless temperature for different dimensionless axial distance for $\overline{{L_{g} }} = 2$, *Ra*_m_ = 2.25

The variation of the dimensionless temperature along a horizontal centerline across the annulus for different Knudsen numbers at *Ra*_m_ = 2.25 and $\overline{{L_{g} }}$ = 2 is illustrated in Fig. 15. The graph shows that there is no temperature jump at the walls for the case of *Kn* = 0. While, as indicated earlier, when Knudsen number increases then the temperature jump increases. Moreover, the figure shows that as *Kn* increases, the gradient of the temperature at the hot boundary decreases, and this leads to the reduction in heat transfers to the annulus regime. This explains the increase of the conduction zone (indicated by linear variation of temperature) as *Kn* increases. The steep nonlinear variation of the temperature close to walls is an indication of the presence of convection heat transfer mechanism. Thus, the dominant mode of heat transfer near the boundaries is convection and away from the boundaries is conduction.

**Fig. 16:**
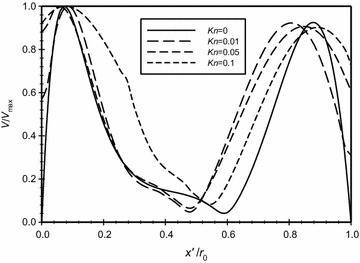
Variation of the dimensionless velocity magnitude for different dimensionless axial distance for $\overline{{L_{g} }} = 3$, *Ra*_m_ = 4.59

For the case where the modified Rayleigh number (*Ra*_m_ = 4.59) and the dimensionless gap spacing between the two cylinders ($\overline{{L_{g} }}$ = 3), Fig. 16 shows the dimensionless velocity variation with the dimensionless axial distance ($\overline{X}$) plotted for different Knudsen numbers. It is very clear from the graph that there is no-slip at the boundaries for the case where *Kn* = 0. The graph shows that as Knudsen number increases then the velocity slip increases at the boundaries. By contrasting Fig. 16 to Fig. 10 where ($\overline{{L_{g} }}$ = 2), the merging of the boundary layers in Fig. 10 is obvious and the effect gets into the core of the flow while for the case of $\overline{{L_{g} }}$ = 3, this effect does not get into the core of the flow where we have much lower velocities between ($x^{\prime}/r_{o} = 0.3{\text{ to }}0.6$).

**Fig. 17:**
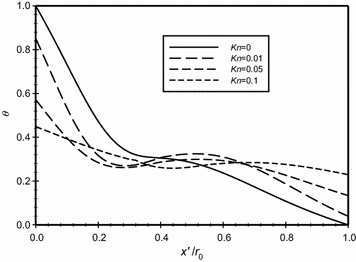
Variation of the dimensionless temperature for different dimensionless axial distance for $\overline{{L_{g} }} = 3$, *Ra*_m_ = 2.58

**Fig. 18:**
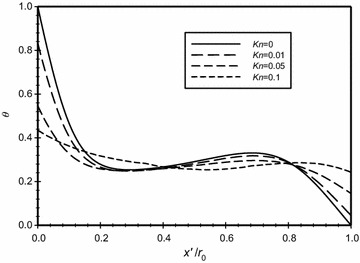
Variation of the dimensionless temperature for different dimensionless axial distance for $\overline{{L_{g} }} = 3$, *Ra*_m_ = 4.59

In Fig. 17, the modified Rayleigh number is *Ra*_m_ = 2.58 and in Fig. 18, this value was fixed to 4.59. The dimensionless gap spacing between the inner and outer cylinders was fixed to ($\overline{{L_{g} }}$ = 3) in the two graphs. The dimensionless temperature distribution with the dimensionless axial distance ($\overline{X}$) is plotted for different Knudsen numbers, the graphs show that as Knudsen number increases then the temperature jump increases due to the rarefaction effects. Also, both graphs show that there is no temperature jump at the boundaries for the case where *Kn* = 0. A comparison of the two figures will show that that the temperature jump for the same Knudsen number at the hot surface for the case of *Ra*_m_ = 2.58 is less than the case where *Ra*_m_ = 4.59 and hence the heat transfer for the larger *Ra*_m_ is higher than the case of lower *Ra*_m_.

**Fig. 19:**
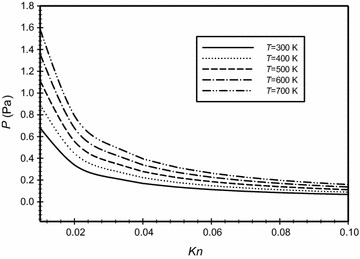
Variation of pressure in the annulus for different Knudsen numbers and different operating temperatures

Figure 19 shows the change of the pressure in the annulus region between the two concentric cylinders as a function of Knudsen number for different operating temperatures. The values are calculated based on the definition of the Knudsen number given in Eq. (1) for air. The graph is a powerful tool that can guide and give valuable information for the manufacturers of the receivers of parabolic trough collectors and the solar evacuated tubes. The graph shows that as the temperature increases then the evacuation pressure increases for the same Knudsen number. The graph also shows that as Knudsen number increases then the evacuation pressure decreases for the same operating temperature. It is obvious from the graph that when the operating pressure in the annulus is in the range of (0.1–1.6 Pa) and the operating temperature is between 300 and 700 K, the flow is in the slip-flow regime. The temperatures range (300–700 K) covers the actual operating range of the PTC’s. For example, if the case of *Kn* = 0.05 and *T* = 300 K and pressure is 0.1357 Pa is taken as a base case. Now, for the same *Kn* but with operating temperature of *T* = 400 K, the resulting pressure is 0.1779. The figure also shows that increasing *Kn* beyond 0.05 within the investigated operating temperatures, the change in the evacuated pressure is small and becomes almost constant compared to the range of *Kn* less than 0.05.

**Fig. 20:**
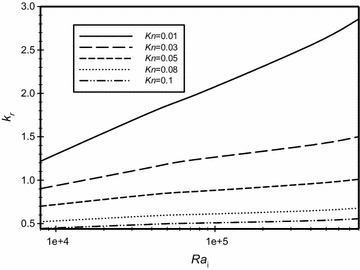
Variation of the conductivity ratio (*K*_r_) in the annulus for different Knudsen numbers (*Kn*) and different Rayleigh numbers (*Ra*_i_) for $\overline{{L_{g} }} = 3$

Finally, the values of the conductivity ratio *k*_r_ with the conventional *Ra*_i_ for different values of *Kn* where $\overline{{L_{g} }}$ = 3 are plotted to facilitate and investigate the effect of using *Ra*_i_ instead of *Ra*_m_. The results are shown in Fig. 20. It is obvious from the graph that the conduction is the dominant mode of heat transfer except for very low Knudsen number (*Kn* = 0.01) where the convection mode of heat transfer is the dominant.

## Conclusions

A steady, two-dimensional analysis of gaseous flow in the annulus region between two concentric cylinders is carried out. This type of flow has a wide variety of applications such as the receiver of the parabolic trough collectors and the evacuated tube collectors. It is found that both the slip velocity and temperature jump increase with increasing Knudsen number (*Kn*). Hence, the rarefication effect results in increase of frictional losses and diminishing of the heat transfer rate that is presented by the thermal conductivity ratio (*k*_r_ = *k*_eff_/*k*) ratio. In addition, it is shown that as the modified Rayleigh number (*Ra*_m_) increases then the heat transfer rate increases and the convection heat transfer mode becomes the dominant mode of heat transfer in the annulus. It is found that there is no consistent effect of the gap spacing on the heat transfer rate in the annulus region between the two concentric horizontal cylinders. A correlation for the thermal conductivity ratio (*k*_r_ = *k*_eff_/*k*) as a function of *Ra*_m_ and *Kn* is introduced.

## List of symbols

*A*_i_: surface area of inner cylinder (m^2^)
C_p_: specific heat (J kg^−1^ K^−1^)
*d*: molecular diameter of the gas (m)
*D*_i_: inner annulus diameter (m)
*D*_o_: outer annulus diameter (m)
*dE*: energy flux on a surface per unit time
*g*: gravity acceleration (m/s^2^)
$$\bar{h}$$
average heat transfer coefficient (W m^−2^ K^−1^)
*k*: thermal conductivity (W m^−1^ K^−1^)
*k*_B_: Boltzman constant = 1.38066 × 10^−23^ J K^−1^
*k*_eff_: effective thermal conductivity (W m^−1^ K^−1^)
*Kn*: Knudsen number
*k*_*r*_: thermal conductivity ratio ($k_{eff} /k_{f}$)
*L*: length of the cylinder (m)
*L*_g_: gap spacing between the two cylinders (*r*_o_ − *r*_i_) (m)
$$\overline{{L_{g} }}$$
dimensionless gap spacing between the two cylinders (*r*_o_ − *r*_i_)/*r*_i_
*Nu*_i_: Nusselt number based on inner cylinder diameter
$$\overline{{Nu_{i} }}$$
average Nusselt number based on inner cylinder diameter
*P*: pressure (Pa)
Pr: Prandtl number
*Q*: heat transfer (W)
*Q*′: heat transfer per unit length (W/m)
*Q*_*cond*_: conductive heat transfer (W)
*R*: gas constant (J kg^−1^ K^−1^)
*r*_i_: inner annulus radius (m)
*r*_o_: outer annulus radius (m)
*Ra*: Rayleigh number [*gβ*(*T*_i_ − *T*_o_)L^3^/*αν*]
*Ra*_c_: Rayleigh number based on *L*_c_ = [*gβ*(*T*_i_ − *T*_o_)*L*_*c*_^3^/*αν*]
*Ra*_i_: Rayleigh number based on the inner diameter
*Ra*_m_: modified Rayleigh number
*T*: temperature (K)
*T*_*c*_: temperature of the first cell from the wall in the computational domain (K)
*T*_i_: temperature of annulus inner surface (K)
*T*_o_: temperature of annulus outer surface (K)
*u*: velocity in x-direction (m/s)
*u*_*c*_: the tangential velocity of the first cell from the wall in the computational domain (m/s)
*v*: velocity in y-direction (m/s)
*V*: velocity magnitude (m/s)
*x, y*: Cartesian coordinates (m)
$$x^{\prime}$$
line, starting from any point a on the inner cylinder and ending at any point b on the outer cylinder (m)
$$\bar{X}$$
dimensionless $$x^{\prime} = {{x^{\prime}} \mathord{\left/ {\vphantom {{x^{\prime}} {r_{o} }}} \right. \kern-0pt} {r_{o} }}$$

### Greek symbols

α: thermal diffusivity (m^2^/s)
β: thermal expansion coefficient (1/*K*)
γ: ratio of the specific heats
λ: molecular mean free path (m)
μ: dynamic viscosity (kg m^−1^ s^−1^)
ν: kinematic viscosity (m^2^ s^−1^)
ρ: density of air, given by ideal gas equation (*P*/R*T*), (kg/m^3^)
σ: Lennard–Jones characteristic length (Å)
σ_T_: thermal accommodation coefficient
σ_v_: momentum accommodation coefficient
*τ*: tangential momentum
θ: dimensionless temperature

### Subscripts

*c*: first cell from the wall in the computational domain
*cond*: conduction
eff: effective
g: gap
i: inner
m: modified
max: maximum
o: outer
r: ratio
*w*: wall
